# Low pre-existing endemic human coronavirus (HCoV-NL63)-specific T cell frequencies are associated with impaired SARS-CoV-2-specific T cell responses in people living with HIV

**DOI:** 10.3389/fimmu.2023.1291048

**Published:** 2024-01-26

**Authors:** Tiza L. Ng’uni, Vernon Musale, Thandeka Nkosi, Jonathan Mandolo, Memory Mvula, Clive Michelo, Farina Karim, Mohomed Yunus S. Moosa, Khadija Khan, Kondwani Charles Jambo, Willem Hanekom, Alex Sigal, William Kilembe, Zaza M. Ndhlovu

**Affiliations:** ^1^ Africa Health Research Institute (AHRI), Nelson R. Mandela School of Medicine, Durban, South Africa; ^2^ Emory-University of Georgia, Center of Excellence of Influenza Research and Surveillance (CEIRS), Lusaka, Zambia; ^3^ Center for Family Health Research in Zambia (CFHRZ), formerly Zambia Emory HIV Research Project (ZEHRP), Lusaka, Zambia; ^4^ Infection and Immunity Research Group, Malawi-Liverpool-Wellcome Trust Clinical Research Programme, Blantyre, Malawi; ^5^ Human Immunodeficiency Virus (HIV) Pathogenesis Program, School of Laboratory Medicine and Medical Sciences, University of KwaZulu Natal, Durban, South Africa; ^6^ Department of Clinical Sciences, Liverpool School of Tropical Medicine, Liverpool, United Kingdom; ^7^ Division of Infection and Immunity, University College London, London, United Kingdom; ^8^ Ragon Institute of Massachusetts General Hospital (MGH), Massachusetts Institute of Technology (MIT) and Harvard University, Cambridge, MA, United States

**Keywords:** HIV, SARS-CoV-2, HCoV-NL63, COVID-19, T-cell response, antibody response

## Abstract

**Background:**

Understanding how HIV affects SARS-CoV-2 immunity is crucial for managing COVID-19 in sub-Saharan populations due to frequent coinfections. Our previous research showed that unsuppressed HIV is associated with weaker immune responses to SARS-CoV-2, but the underlying mechanisms are unclear. We investigated how pre-existing T cell immunity against an endemic human coronavirus HCoV-NL63 impacts SARS-CoV-2 T cell responses in people living with HIV (PLWH) compared to uninfected individuals, and how HIV-related T cell dysfunction influences responses to SARS-CoV-2 variants.

**Methods:**

We used flow cytometry to measure T cell responses following PBMC stimulation with peptide pools representing beta, delta, wild-type, and HCoV-NL63 spike proteins. Luminex bead assay was used to measure circulating plasma chemokine and cytokine levels. ELISA and MSD V-PLEX COVID-19 Serology and ACE2 Neutralization assays were used to measure humoral responses.

**Results:**

Regardless of HIV status, we found a strong positive correlation between responses to HCoV-NL63 and SARS-CoV-2. However, PLWH exhibited weaker CD4^+^ T cell responses to both HCoV-NL63 and SARS-CoV-2 than HIV-uninfected individuals. PLWH also had higher proportions of functionally exhausted (PD-1high) CD4^+^ T cells producing fewer proinflammatory cytokines (IFNγ and TNFα) and had elevated plasma IL-2 and IL-12(p70) levels compared to HIV-uninfected individuals. HIV status didn’t significantly affect IgG antibody levels against SARS-CoV-2 antigens or ACE2 binding inhibition activity.

**Conclusion:**

Our results indicate that the decrease in SARS-CoV-2 specific T cell responses in PLWH may be attributable to reduced frequencies of pre-existing cross-reactive responses. However, HIV infection minimally affected the quality and magnitude of humoral responses, and this could explain why the risk of severe COVID-19 in PLWH is highly heterogeneous.

## 1 Introduction

The novel Severe Acute Respiratory Syndrome Coronavirus 2 (SARS-CoV-2), responsible for causing coronavirus disease 2019 (COVID-19), has emerged as a significant public health menace, leading to the unprecedented loss of millions of lives globally (1). The World Health Organization (WHO) designated it as a pandemic on March 11^th^, 2020 (2). It is hypothesized that the rapid increase in global cases primarily resulted from a lack of pre-existing immunity to the novel SARS-CoV-2 (3). While many countries have successfully curbed the COVID-19 pandemic through extraordinary preventive measures, the potential for a global resurgence still looms large, hence the need to better understand immune responses to SARS-CoV-2, particularly in regions with a high HIV burden like sub-Saharan Africa.

To design the next generation of COVID-19 vaccines, it is essential to better understand both individual and population-level immunity, encompassing both humoral and adaptive responses (3). While the role of antibodies in clearing the virus and influencing the severity of COVID-19 is relatively well understood, the understanding of T cell immunity to SARS-CoV-2 has been limited due to a lack of studies focusing on T cells (4).

Furthermore, understanding the potential susceptibility of People Living with HIV (PLWH) to SARS-CoV-2 infection and severe COVID-19 holds significant relevance for developing next generation vaccines and therapies. Although it is well known that HIV weakens the immune system, which could have a negative impact on SARS-CoV-2 immunity, the precise immune defects associated with reduced SARS-CoV-2 immune responses in PLWH are still unresolved (5–7). As the COVID-19 pandemic is still rapidly evolving, more studies are needed to understand the interplay between HIV and SARS-CoV-2 in PLWH to inform both clinical and public health guidelines on HIV and SARS-CoV-2 coinfection.

It has been shown that over 90% of the human population is seropositive for at least three of the endemic human coronaviruses (EHC), HCoV-OC43, HCoV-HKU1, HCoV-NL63 and HCoV-229E, which widely circulate in the human population (8, 9). The memory T cell responses to these EHCs commonly exhibit cross-reactivity with SARS-CoV-2 (10, 11). In fact, detectable SARS-CoV-2-specific T cells have been identified in some individuals who lack any prior history of COVID-19 or SARS-CoV-2 exposure from an infected person (9, 12, 13). However, the potential influence of HIV infection on these cross-reactive immune responses remains under explored.

In this study, we utilized cohorts comprising both HIV-infected and HIV-uninfected participants from South Africa and Zambia who had contracted COVID-19 to explore the mechanisms linked to impaired SARS-CoV-2-specific T cell responses in PLWH. We examined whether pre-existing immunity to EHCs, specifically HCoV-NL63, influence SARS-CoV-2-specific T cell immunity and humoral responses in PLWH. Our findings demonstrate that HIV infection HIV exerts a more pronounced impact on T cell responses to both HCoV-NL63 and SARS-CoV-2, but minimally impacted humoral immunity against SARS-CoV-2.

## 2 Materials and methods

### 2.1 Ethical statement

The study was approved by the University of KwaZulu-Natal Institutional Review Board (approval BREC/00001275/2020) and the National Health Research Authority and University of Zambia Biomedical Research Ethics Committee (REF. No. 1648-2021). Adult patients (18 years and older) presenting at King Edward VIII, Inkosi Albert Luthuli Central, Kwadabeka community healthcare center or Clairwood Hospitals in Durban, South Africa, and the Center for Family Health Research in Zambia, research clinics and collaborating GRZ health facilities in Lusaka and Ndola, between October 2020 to August 2021, diagnosed to be SARS-CoV-2 PCR positive were eligible for the study. All participants enrolled into the study provided written informed consent.

### 2.2 Study participants and sample collection

A total of 68 adult participants were recruited in South Africa and Zambia (34 from each site). We also recruited 11 healthy controls who were HIV-uninfected and matched for sex and age to the South African cohort (**Table 1**). The groups are represented as HIV-uninfected and SARS-CoV-2-infected (HIV-/SARS-CoV-2+); HIV-infected and SARS-CoV-2-infected (HIV+/SARS-CoV-2+); and healthy controls (HIV-/SARS-CoV-2-). Peripheral blood mononuclear cells (PBMCs) and plasma samples collected from Zambia were shipped to Durban, South Africa and matched with samples collected from South Africa. The matching criteria included HIV status, age, sex, time point and the wave of infection (wild-type, beta, or delta infection). PBMCs and plasma samples used in this study were collected between 1- and 4-weeks post-SARS-CoV2 PCR-positive diagnosis. Peripheral blood mononuclear cells (PBMCs) from healthy donors collected for other studies before 2018, prior to the COVID-19 pandemic, were included as healthy controls.

**Table 1:** Cohort Demographics and Clinical Characteristics.

	All (N = 79)	HIV- uninfected (*n* = 48)	HIV-infected (*n* = 20)	Healthy controls (*n* = 11)	Statistics
Demographics					
**Age years, median (IQR)**	35 (27 – 44)	34.5 (28 - 43.75)	41.5 (32 - 49.75)	19.5 (18.75 – 21.50)	0.0002^a^
**Male sex, n (%)**	29 (36.71)	22 (45.83)	4 (20)	3 (27.27)	ns^b^
**Female sex, n (%)**	50 (63.29)	26 (54.17)	16 (80)	8 (72.73)	ns^b^
**Days since diagnosis, median (range)**	14 (1 – 28)	14 (6 – 28)	13.5 (7 – 28)	–	ns^c^
HIV-associated parameters					
**HIV viral load copies/mL, median (IQR)**		–	13,981 (352-65,386)	–	n/a
**CD4 cells/µL median (IQR)**	760.5 (580.5 - 874.3)	783 (633 -921.5)	197 (75.5 - 726)	840 (739 - 996.3)	ns^d^
Disease severity					
**Asymptomatic, n (%)**	11 (16.18)	10 (20.83)	1 (5)	–	ns^b^
**Mild, n (%)**	54 (79.4)	37 (77.1)	17 (85)	–	ns^b^
**Severe/oxygen supplementation, n (%)**	3 (4.41)	1 (2.08)	2 (10)	–	ns^b^

P values were calculated by one-way ANOVA, Fischer’s exact test, Mann-Whitney test or Kruskal-Wallis test for unpaired three groups. Convalescent HIV-infected and HIV-uninfected individuals were infected with either the beta (second wave) or delta (third wave) variants.

a One-way ANOVA,

b Fisher’s exact probability test.

c Mann-Whitney test.

d Kruskal-Wallis test.

‘ns’ not significant and ‘n/a’ not applicable.

### 2.3 Peptide pools

To detect virus-specific T-cell responses, PBMCs were stimulated with the following peptide pools: 1) PepMix™ SARS-CoV-2 (Spike B.1.351/Beta Variant): Pool of 315 peptides derived from a peptide scan (15mers with 11 aa overlap) through the entire Spike glycoprotein - containing mutations D0080A, D0215G, L0242-, A0243-, L0244-, K0417N, E0484K, N0501Y, D614G and A0701V (JPT Peptide Technologies). 2) PepMix™ SARS-CoV-2 (Spike B.1.617.2/Delta Variant): Pool of 315 peptides derived from a peptide scan (15mers with 11 aa overlap - 4x 13mer + last peptide = 17mer) through the Spike glycoprotein - containing mutations T0019R, G0142D, E0156-, F0157-, R0158G, L0452R, T0478K, D0614G, P0681R and D0950N (JPT Peptide Technologies). 3) PepMix™ HCoV-NL63 (Spike Glycoprotein): Pool of 337 peptides derived from a peptide scan (15mers with 11 aa overlap) through the Spike glycoprotein (Swiss-Prot ID: Q6Q1S2) of Human Coronavirus (HCoV) (JPT Peptide Technologies). 4) PepTivator SARS-CoV-2 Prot_S Complete: Pool of peptides consisting mainly of 15-mers overlapping by 11 amino acids residues covering the entire protein coding sequence of the spike glycoprotein (aa 5–1273) (Miltenyi Biotec). 5) PepTivator SARS-CoV-2 Prot_S1: Pool of peptides covering the N-terminal S1 domain of the spike glycoprotein (aa 1–692) (Miltenyi Biotec).

### 2.4 T cell phenotyping by flow cytometry

PBMCs were isolated from blood samples by density gradient method and cryopreserved in liquid nitrogen prior to being used for flow cytometry. Frozen PBMCs were thawed, rested, and stimulated for 18 h at 37°C, 5% CO2 with the following peptide pools: SARS-CoV-2 S and S1 (wild-type – WT, 2 µg/ml) (Miltenyi, Biotec), beta and delta variants (0.5 µg/ml) (JPT Peptide Technologies), or HCoV-NL63 (0.5 µg/ml) (JPT Peptide Technologies). Staphylococcal enterotoxin B (SEB, 0.5 µg/ml) was used as a positive control. Unstimulated wells were also included as negative controls. Brefeldin A (BioLegend, CA) and CD28/CD49d (BD Biosciences, Franklin Lakes, NJ) were added ahead of the 18 h incubation at 5 and 1 µg, respectively. The cells were stained with an antibody cocktail containing: Live/Dead fixable aqua dead cell stain, anti-CD3 PE-CF594 (BD), anti-CD4 Brilliant Violet (BV) 650, anti-CD8 BV 786 (BD), anti-CD38 Alexa Fluor (AF) 700 (BD), anti-human leukocyte antigen (HLA) – DR Allophycocyanin (APC) Cy 7 (BD), and anti-programmed cell death protein 1 (PD) BV 421 (BD). After a 20-minute incubation at room temperature, the cells were washed, fixed, and permeabilized using the BD Cytofix/Cytoperm fixation permeabilization kit. Thereafter, the cells were stained for 40 minutes at room temperature with an intracellular antibody cocktail containing: anti-IFN-γ BV 711 (BD), anti-IL-2 PE (BD), anti-TNF-α PE-Cy 7 and anti-granzyme B PE-CF594 (BD). Finally, the cells were washed and acquired on an LSR Fortessa and analyzed on FlowJo v10.7.1. Differences between groups were significant at a p< 0.05. Statistical analyses were performed using GraphPad Prism 9.0 (GraphPad Software, Inc, San Diego, CA).

### 2.5 Cytokine and chemokine measurements

The following cytokines and chemokines, IL-1β, IL-1ra, IL-2, IL-4, IL-6, IL-7, IL-8, IL-9, IL-10, IL-12p70, IL-13, IL-15, IL-17, basic FGF, eotaxin, granulocyte-colony stimulating factor (G-CSF), granulocyte macrophage-colony stimulating factor (GM-CSF), IFN-γ, interferon gamma-induced protein-10 (IP-10), monocyte chemoattractant protein-1 (MCP-1), macrophage inflammatory protein 1 (MIP-1)α, MIP-1β, platelet-derived growth factor- (PDGF-) BB, regulated on activation, normal T cell expressed and secreted (RANTES), TNF-α, and vascular endothelial growth factor (VEGF), were simultaneously assessed in plasma samples from healthy controls and HIV-infected and HIV-uninfected COVID-19 participants via the Bio-Plex Pro Human Cytokine 27-plex Assay (Bio-Rad) as per manufacturer’s instructions. Briefly 50 μL of plasma samples and various concentrations of the assay standards were added in duplicate to a 96-well plate containing magnetic beads. The plate was incubated for 30 minutes followed by a wash step. The plate was subsequently coated with biotinylated detection antibody solution and incubated for a further 30 minutes. After the 30-minute incubation, the plate was washed and streptavidin-conjugated phycoerythrin was added to the plate and incubated for 10 minutes. After this final incubation, the plate was washed, and assay buffer was added to each well. Data was acquired using the Bio-Plex Array Reader system 2200 (Bio-Rad). A standard curve was derived using the different concentrations of the assay standards. All plasma samples from participants were assayed on the same plate at the same time in duplicate. Intra-assay variability was represented as the coefficient of variation as per manufacturer’s instructions.

### 2.6 V-PLEX COVID-19 serology assay

The MSD V-PLEX COVID-19 Serology platform was used to quantitatively measure antibodies to SARS-CoV-2 antigens including its variants. The kits comprise 96-well plates with antigens precoated to individual carbon spots. Each well on the 96-well plate contains eight SARS-CoV-2-related antigens coated at the bottom of the well. The assay was performed as previously described (14, 15) and per manufacturer’s instructions. Briefly, to measure antigen-specific IgG antibodies, plates were blocked with MSD Blocker A (150 μL/well) after which reference standard, controls and samples diluted to 1:50000 in Diluent buffer were added. After incubation, detection antibody (MSD SULFO-TAG™ Anti-Human IgG Antibody) diluted to 2 μg/mL in Diluent 100 (MSD) was used to label bound antibodies at 50 μL/well. This was followed by the addition of 150 μL MSD GOLD™ Read Buffer B and plates were read using an MSD QuickPlex SQ120 instrument. Calibration curves were used to calculate antibody concentrations and were established by fitting the signals from the calibrators to a 4-parameter logistic (or sigmoidal dose-response) model. Best quantification of unknown samples was achieved by generating a calibration curve for each plate using a minimum of two replicates at each calibrator level. Antibody unit concentrations in controls and diluted samples were determined from their ECL signals by backfitting to the calibration curve. Quantification was reported in Arbitrary Units/mL (AU/mL).

### 2.7 V-PLEX COVID-19 ACE2 neutralization assay

The V-PLEX COVID-19 ACE2 Neutralization Kit was used to measure antibodies that block the binding of angiotensin-converting enzyme 2 (ACE2) to the SARS-CoV-2 Spike and RBD antigens, including variants of the SARS-CoV-2 virus. The assay serves as a high-throughput alternative to traditional neutralization assays. Plates are provided with antigens on spots in the wells of a 96-well plate. Blocking antibodies in the samples bind to antigens on the spots, and human ACE2 protein conjugated with MSD SULFO-TAG is used for detection. The assay was performed according to manufacturer’s instructions. Briefly, 150 µL/well of Blocker A solution was added to the plates, sealed with an adhesive and incubated at RT with shaking (~700 rpm) for 30 minutes. The plates were washed three times with 150 µL/well of 1X MSD Wash buffer. Samples were prediluted (1:10 dilution) according to the manufacturer’s instructions. Samples and calibrators were then added to the plate (25 µL/well), plates sealed with an adhesive plate seal and incubated at RT with shaking (~700 rpm) for 1h. After the incubation, 25 µL/well of 1X SULFO-TAG Human ACE2 Protein detection solution was added to the plate. The plates were sealed with an adhesive plate seal and incubated at RT with shaking (~700 rpm) for 1h. After the detection incubation step, the plates were washed three times with 150 µL/well of 1X MSD Wash buffer. MSD GOLD Read Buffer B was added (150 µL/well) and the plate was immediately read on the MSD instrument. Results were reported as percent inhibition, calculated using the equation below. Percentage inhibition was calculated relative to the assay calibrator (maximum 100% inhibition). Highly positive samples show high percent inhibition whereas negative or low samples show low percent inhibition.


$$\%\text{Inhibition}=1-\frac{\text{Average Sample ECL Signal}}{\text{Average ECL Signal of Calibrator }8\;(\text{Diluent only})}\times 100$$

### 2.8 Enzyme-linked immunosorbent assay

This in-house developed indirect qualitative ELISA was used to measure human IgG specific for the SARS-CoV2 RBD antigen in human plasma. To set up our in-house quantitative ELISA for COVID-19 serology, standard techniques were applied. Briefly, 96-well plates were coated with 100 µL/well of 1ug/mL Recombinant RBD (diluted in 1x PBS) except for blank and the “No RBD” negative control wells. blank and the ‘No RBD’ negative control wells contained 100 µL 1x PBS only. Plates were covered and incubated overnight at 4°C. The coated ELISA plates were washed four times with Wash buffer (containing 1x PBS and 0.05% Tween-20). The plates were then blocked with 200 µL/well of ELISA buffer (containing 1x PBS, 1% BSA and 0.05% Tween-20), except for the blank wells which contained 200 µL/well of Wash buffer. The plates were covered and incubated for 2h. After incubation, 100 µL/well of diluted samples (1:200 dilution in ELISA buffer) and positive controls were added to appropriate wells. To the “No RBD”, blank, and “No primary antibody” wells, 100 µL/well of 0.2 µg/mL standard, Wash buffer and ELISA buffer were added, respectively. The plates were covered and incubated for 90 minutes at RT. Following incubation, the plates were washed four times with Wash buffer and 100 µL/well of peroxidase-conjugated anti-human IgG (1:2000) was added to the wells except the blank wells to which 100 µL/well Wash buffer was added. The plates were then covered and incubated for 60 minutes at RT. The plates were washed four times with Wash buffer and once with 1x PBS. The plates were tapped to remove residual PBS and 100 µL of developing buffer added to all wells. The plates were incubated for 5 minutes at RT and the reaction was stopped by adding 100 µL of 1N HCl to each well. The plates were read immediately at an optical density (OD) of 490 using an ELISA reader. The ELISA controls and test sample result OD values were blank corrected before interpretation. The ELISA test was negative if the average OD value was less than or equal to 0.7 and positive if the average OD value was greater than 0.7.

### 2.9 Statistical analyses

Prism 9 (GraphPad Software) was used for statistical analysis as follows: the two-tailed Mann–Whitney U test was used for single comparisons of independent groups, the Wilcoxon-test paired t test was used to compare two paired groups. For multiple groups statistical significance was assessed using a one-way analysis of variance (ANOVA) with multiple comparisons. The non-parametric Spearman test was used for correlation analysis. The statistical significances are indicated in the figures (*p< 0.05, **p< 0.01, ***p< 0.001, and ****p< 0.0001) and all tests were two-tailed.

## 3 Results

### 3.1 PLWH have lower frequencies of HCoV-specific CD4^+^ T cells compared to HIV-uninfected individuals

The impact of pre-existing immune responses against endemic human coronaviruses (EHC), particularly HCoV-NL63, on SARS-CoV-2 specific T cell responses has been a subject of interest. Previous studies have linked existing cross-reactive memory responses to EHC with milder COVID-19 outcomes and robust vaccine responses, but how these responses are affected in PLWH remains unclear (9, 10, 16). Thus, our investigation aimed to determine if HIV infection alters the influence of pre-existing EHC responses on SARS-CoV-2 specific T cell responses.

**Figure 1:**
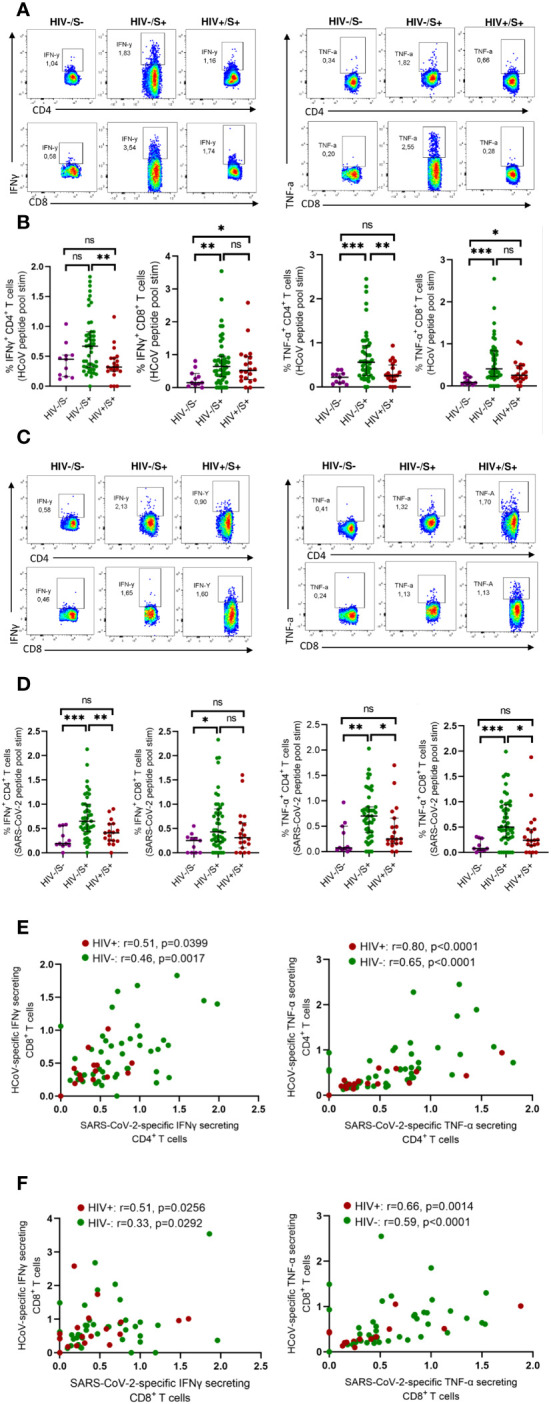
Comparison of HCoV-specific T cells in convalescent HIV-infected and HIV-uninfected individuals and healthy controls. Blood samples collected between 1- and 28-days post infection during the second (beta) and third (delta) waves were used. Intracellular cytokine staining (ICS) was performed to detect cytokine-producing T cells to HCoV overlapping peptide pools in HIV-uninfected (HIV-/SARS-CoV-2+, *n* = 47) and PLWH (HIV+/SARS-CoV-2+, *n* = 20) individuals and healthy controls (HIV-/SARS-CoV-2-; HC, *n* = 11). **(A)** Representative flow cytometry plots for the identification of antigen-specific CD4^+^ and CD8^+^ T cells based on expression IFNγ and TNF-α, following 18-h stimulation with HCoV peptides pools. **(B)** Summary plots showing the frequency of HCoV-specific CD4^+^ and CD8^+^ T cells (IFNγ^+^ and TNF-α^+^). **(C)** Representative flow cytometry plots for the identification of antigen-specific CD4^+^ and CD8^+^ T cells based on expression of IFNγ and TNF-α, following 18-h stimulation with SARS-CoV-2 peptides pools. **(D)** Summary plots showing the frequency of SARS-CoV-2-specific CD4^+^ and CD8^+^ T cells (IFNγ^+^ and TNF-α^+^). **(E)** Correlation of HCoV-specific and SARS-CoV-2-specific CD4^+^ T cells in HIV-infected and HIV-uninfected individuals based on expression of IFNγ and TNF-α. **(F)** Correlation of HCoV-specific and SARS-CoV-2-specific CD8^+^ T cells in HIV-infected and HIV-uninfected individuals based on expression of IFNγ and TNF-α. Significance was determined by two-tailed Mann-Whitney test and the two-tailed nonparametric Spearman test was used for correlation analysis, *p*< 0.05 was considered statistically significant. *p< 0.05, **p< 0.01, ***p< 0.001. ‘ns’ not significant.

Initially, we assessed the frequency of T cells reactive to HCoV-NL63 in COVID-19 convalescent individuals, with and without HIV infection. For comparison, we included HIV-uninfected individuals sampled before the pandemic as healthy controls. To gauge pre-existing immune responses to the endemic human coronavirus, we employed peptide pools encompassing the entire Spike Glycoprotein of HCoV-NL63. This choice was informed by HCoV-NL63 being a prominent endemic human coronavirus circulating in the region (8). PBMCs were stimulated with HCoV-NL63 peptide pools and responding cytokine-producing cells enumerated as described in the methods. Our findings, illustrated through representative flow plots (**Figure 1A**) and collated data (**Figure 1B**), indicate that a considerable portion of the participants exhibited visible HCoV-NL63-specific CD4^+^ T and CD8^+^ T cell responses, as signified by the presence of IFN-γ or TNF-a producing cells. Notably, the aggregate data demonstrates that HIV-uninfected individuals with COVID-19 displayed elevated levels of HCoV-NL63 specific CD4^+^ T cells compared to PLWH and healthy controls (**Figure 1B**). The stable and sustained HCoV-NL63 responses observed in HIV-uninfected individuals are likely due to a relatively stable pool of HCoV-NL63-specific memory CD4^+^ T cells. Our findings also showed significantly higher frequencies of SARS-CoV-2-specific CD4^+^ and CD8^+^ T cells following stimulation with SARS-CoV-2 peptide pools shown in the representative flow plots and summary plots (**Figures 1C, D**). These observations underscore the potential of SARS-CoV-2 to activate HCoV-NL63 specific CD4^+^ T cells and emphasize the attenuation of pre-existing memory responses due to the presence of HIV.

Next, we measured SARS-CoV-2 specific T cell responses. We found a correlation between HCoV-NL63 -specific and SARS-CoV-2-specific CD4^+^T and CD8^+^ T cell responses regardless of HIV status (**Figures 1E, F**). These results are consistent with a previous report (17) that showed pre-existing cross-reactive CD4^+^ T cells enhance immune responses to SARS-CoV-2 infection and BNT162b2 vaccination (17).

### 3.2 Altered phenotypic characteristics of bulk CD4^+^ T cells in PLWH

**Figure 2:**
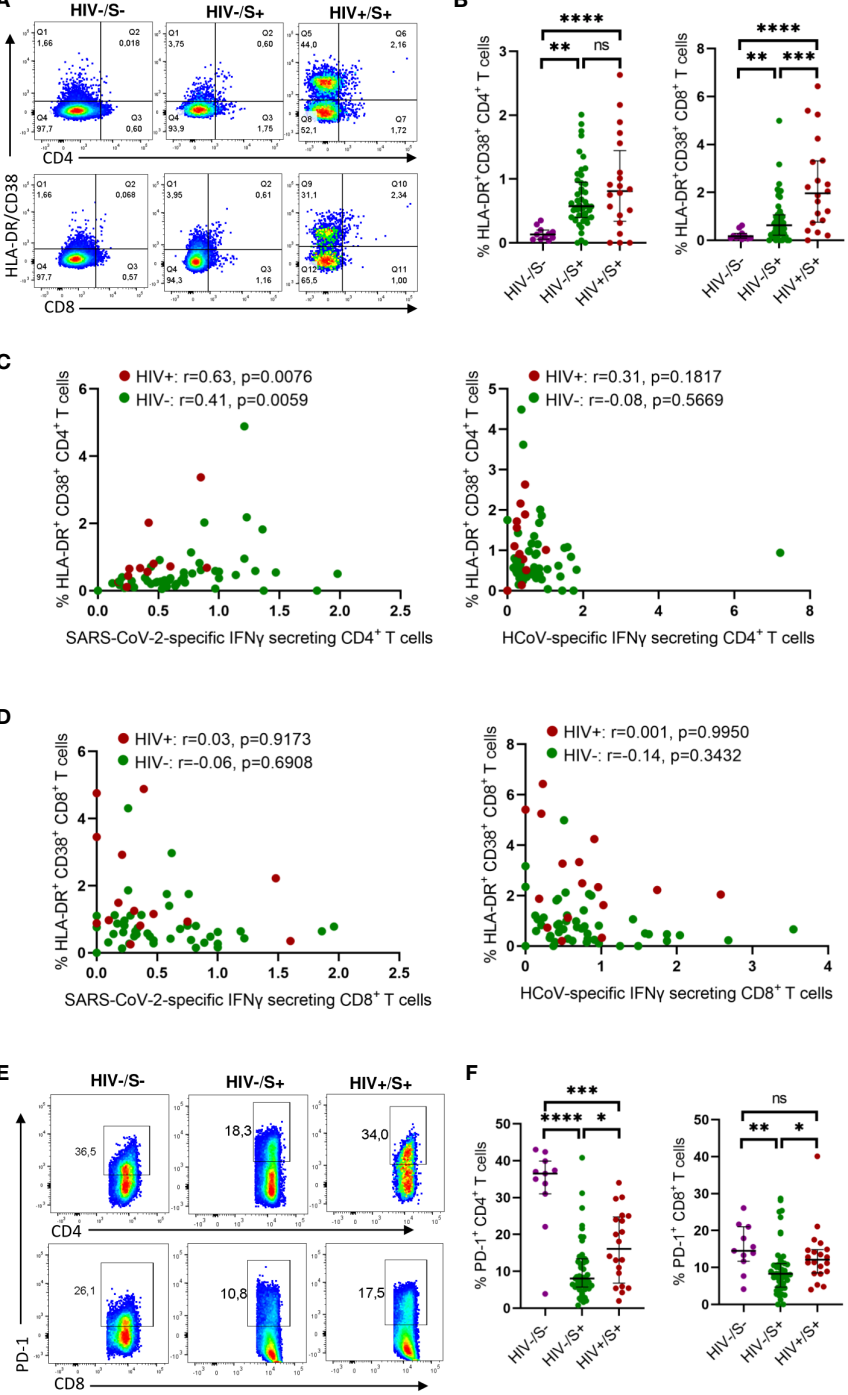
Comparison of the activation and exhaustion profile of HCoV-specific CD4^+^ and CD8^+^ T cells in COVID-19 convalescent HIV-infected and HIV-uninfected Individuals and healthy controls. Blood samples collected between 1- and 28-days post infection during the second (beta) and third (delta) waves were used to detect activated and exhausted T cells in HIV-uninfected (HIV-, *n* = 46), PLWH (HIV+, *n* = 20) and healthy controls (*n* = 11). **(A)** Representative flow cytometry plots for the identification of activated (HLA-DR^+^CD38^+^) CD4^+^ and CD8^+^ T cells. **(B)** Summary plots of the frequency of activated CD4^+^ and CD8^+^ T cells based on the expression of HLA-DR, CD38. Correlation of T cell activation of SARS-CoV-2 and HCoV-specific **(C)** CD4^+^ and **(D)** CD8^+^ T cells in HIV-infected and HIV-uninfected individuals. **(E)** Representative flow cytometry plots and **(F)** summary data for the identification of exhausted (PD-1^+^) CD4^+^ and CD8^+^ T cells. Significance was determined by two-tailed Mann-Whitney test and these two-tailed nonparametric Spearman test was used for correlation analysis, *p*< 0.05 was considered statistically significant. *p< 0.05, **p< 0.01, ***p< 0.001, ****p< 0.0001. ‘ns’ not significant.

To investigate the mechanisms associated with impaired T cell response in PLWH, we first compared the activation profile of CD4^+^ and CD8^+^ T cells between PLWH and HIV-uninfected COVID-19 convalescent individuals as well as healthy controls. Here, T cell activation is defined as HLA-DR^+^CD38^+^ T cells. Representative flow plots (**Figure 2A**) and aggregate data (**Figure 2B**) show that PLWH had greater frequencies of activated (HLA-DR^+^ CD38^+^) CD8^+^ T cells (*p* = 0.0006) and a trend towards more activated CD4^+^ T cells compared to HIV-uninfected individuals and healthy controls (**Figure 2B**). Furthermore, we show that both the CD4^+^ and CD8^+^ T cells were more activated in the HIV-infected and HIV-uninfected COVID-19 participants compared to the healthy control group (**Figure 2B**).

Our investigation of the potential impact of T cell activation on the frequency of SARS-CoV-2 and HCoV-NL63 specific responses unveiled a significant correlation between the T cell activation and the frequency of SARS-CoV-2 CD4^+^ T cell responses, irrespective of HIV status (PLWH: *p*=0.0076 and HIV-negative individuals: *p*=0.0059) (**Figure 2C**). These findings indicate that the SARS-CoV-2 specific CD4^+^ T cells represented recent activated effector cells. Conversely, no discernible correlation was detected between the frequency of HCoV-NL63 responses and T cell activation (**Figure 2C**), which could be attributed to HCoV-NL63 responses being quiescent long-lived memory cells. Moreover, no correlation was observed for CD8^+^T cell responses (**Figure 2D**).

Next, we examined T cell exhaustion, which we defined by the presence of the canonical T cell exhaustion marker PD-1 (18). Surprisingly, the healthy controls had significantly higher PD-1 expression in CD4^+^ T cells compared to the HIV-infected and HIV-uninfected groups whereas the PD-1 expression in CD8 T cells was only significantly higher in the HIV-uninfected group (**Figure 2D**). This could probably be due to other underlying conditions or infections. However, our observations revealed that PLWH exhibited higher frequencies of exhausted CD4^+^ and CD8^+^ T cells (*p* = 0.0149 for CD4^+^ T cells and *p* = 0.0105 for CD8^+^ T cells, respectively) as depicted in representative plots and aggregate data (**Figures 2E, F**). These findings are line with previous reports (19–22). However, we did not find any significant correlation between either HCoV-NL63 or SARS-CoV-2 responses and T cell exhaustion (**Supplementary Figure 1**). Taken together, these results suggest a link between weakened SARS-CoV-2-specific responses and heightened T-cell activation.

### 3.3 Elevated plasma cytokine levels persist during recovering COVID-19 patients

**Figure 3:**
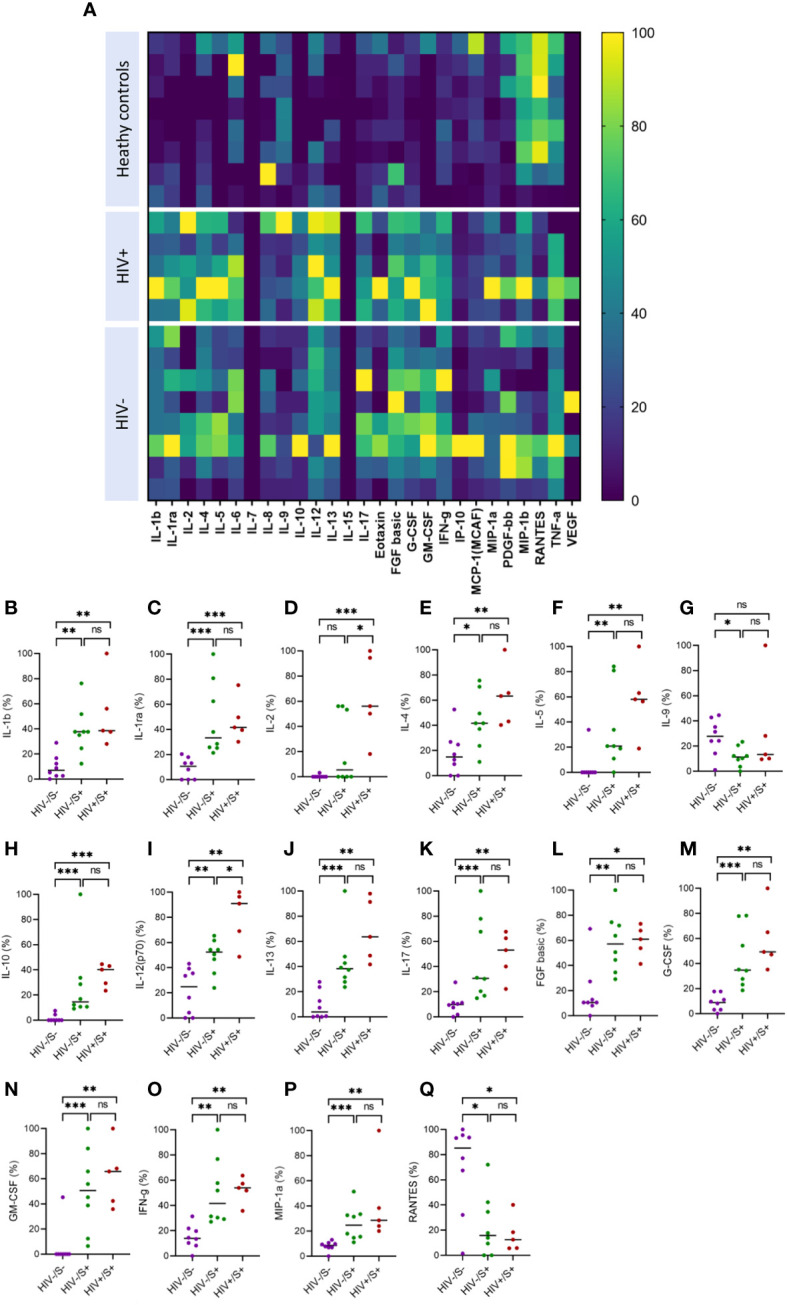
Comparison of plasma cytokine and chemokine levels in cells in convalescent HIV-infected and HIV-uninfected individuals and healthy controls. Serum samples collected between 1- and 28-days post infection were used to measure cytokine and chemokine levels by the Bio-Plex assay. **(A)** Heatmap showing normalized cytokine and chemokine levels (in percentages) in convalescent HIV-infected (HIV+/S+, *n* = 5) and HIV-uninfected (HIV-/S+, *n* = 8) individuals and healthy controls (HIV-/S-, *n* = 8). Summary plots of **(B)** IL-1b, **(C)** IL-1ra, **(D)** IL-2, **(E)** IL-4, **(F)** IL-5, **(G)** IL-9, **(H)** IL-10, **(I)** IL-12(p70), **(J)** IL-13, **(K)** IL-17, **(L)** FGF basic, **(M)** G-CSF, **(N)** GM-CSF, **(O)** IFN-g, **(P)** MIP-1a and **(Q)** RANTES. Significance was determined by two-tailed Mann-Whitney test, *p*< 0.05 was considered statistically significant. *p< 0.05, **p< 0.01, ***p< 0.001. ‘ns’ not significant.

Systemic inflammation has been shown to impair immune responses to SARS-CoV-2 infection (23, 24). To investigate whether systemic inflammation contributes to impaired T cell function observed in PLWH, we used the Bio-Plex Pro Human Cytokine Grp I Panel 27-Plex Assay to measure circulating chemokines and cytokines in COVID-19 convalescent patients and healthy controls. This multiplex analysis allowed us to measure 27 plasma cytokines produced in the convalescent phase of infection. The groups were denoted as HIV-uninfected and SARS-CoV-2-infected (HIV-/S+); HIV-infected and SARS-CoV-2-infected (HIV+/S+); and healthy controls being HIV-uninfected and SARS-CoV-2-uninfected (HIV-/S-). 21 participants comprising of 8 healthy controls (HIV-/S-); 5 HIV+/S+; 8 HIV-/S+ selected based on sample availability were used for these studies (**Table 2**). The plasma cytokine concentrations from the Luminex readout were normalized and presented as percentages, where 0% defined the smallest mean in each data set and 100% defined the largest mean in each data set shown as heatmap (**Figure 3A**). Our data show that the convalescent phase of SARS-CoV-2 infection is associated with persistent cytokine storm including IL-1b, IL-1ra, IL-2, IL-4, IL-5, IL-9, IL-10, IL-12(p70), IL-13, IL17, FGF basic, G-CSF, GM-CSF, IFN-g, MIP-1a and RANTES (**Figures 3B–Q**). Among COVID-19 donors, IL-2, and IL-12(p70) were significantly elevated in PLWH compared to HIV-uninfected participants (**Figures 3D, I**). Additionally, IL-10 trended toward higher levels in PLWH relative to HIV-uninfected individuals and healthy controls (**Figure 3H**). However, we did not find significant differences in the levels of IL-6, IL-8, Eotaxin, IP-10, MCP-1(MCAF), PDGF-bb, MIP-1b and TNF-a in the three groups (**Supplementary Figure 2**). Overall, these data show that convalescent COVID-19 donors had elevated systemic inflammation as widely reported in the literature (25–27). Importantly, HIV infection was associated with significantly elevated cytokines such as IL-2, IL-12p70, and a trend towards more IL-10. These three cytokines have previously been associated with severe COVID-19 disease (28, 29). Overall, the plasma cytokine/chemokine levels in COVID-19 participants were much higher than in healthy donors in the convalescent phase of infection.

**Table 2:** Description of the subset of samples used to measure cytokine levels and antibody responses.

	Healthy controls (*n* = 8)	HIV-uninfected (*n* = 8)	HIV-infected (*n* = 6)
**Age mean (range)**	22.4 (18 – 33)	31.8 (20 – 40)	44.4 (25 – 65)
**Male, n (%)**	2 (25)	5 (62.5)	1 (20)
**Female, n (%)**	6 (75%)	3 (37.5)	4 (80)
**Days since diagnosis, meadian (range)**	–	7 (7 – 21)	14 (7 – 22)
**CD4 count, median (range)**	840 (634 – 1348)	819 (624 – 1131)	260 (133 – 566)
**SARS-CoV-2-specific responses**	No	Yes	Yes
**HCoV-specific responses**	Yes	Yes	Yes

Convalescent HIV-infected and HIV-uninfected individuals were infected with either the beta (second wave) or delta (third wave) variants.

### 3.4 Anti-RBD IgG levels do not correlate with SARS-CoV-2-specific CD4^+^T cell responses

**Figure 4:**
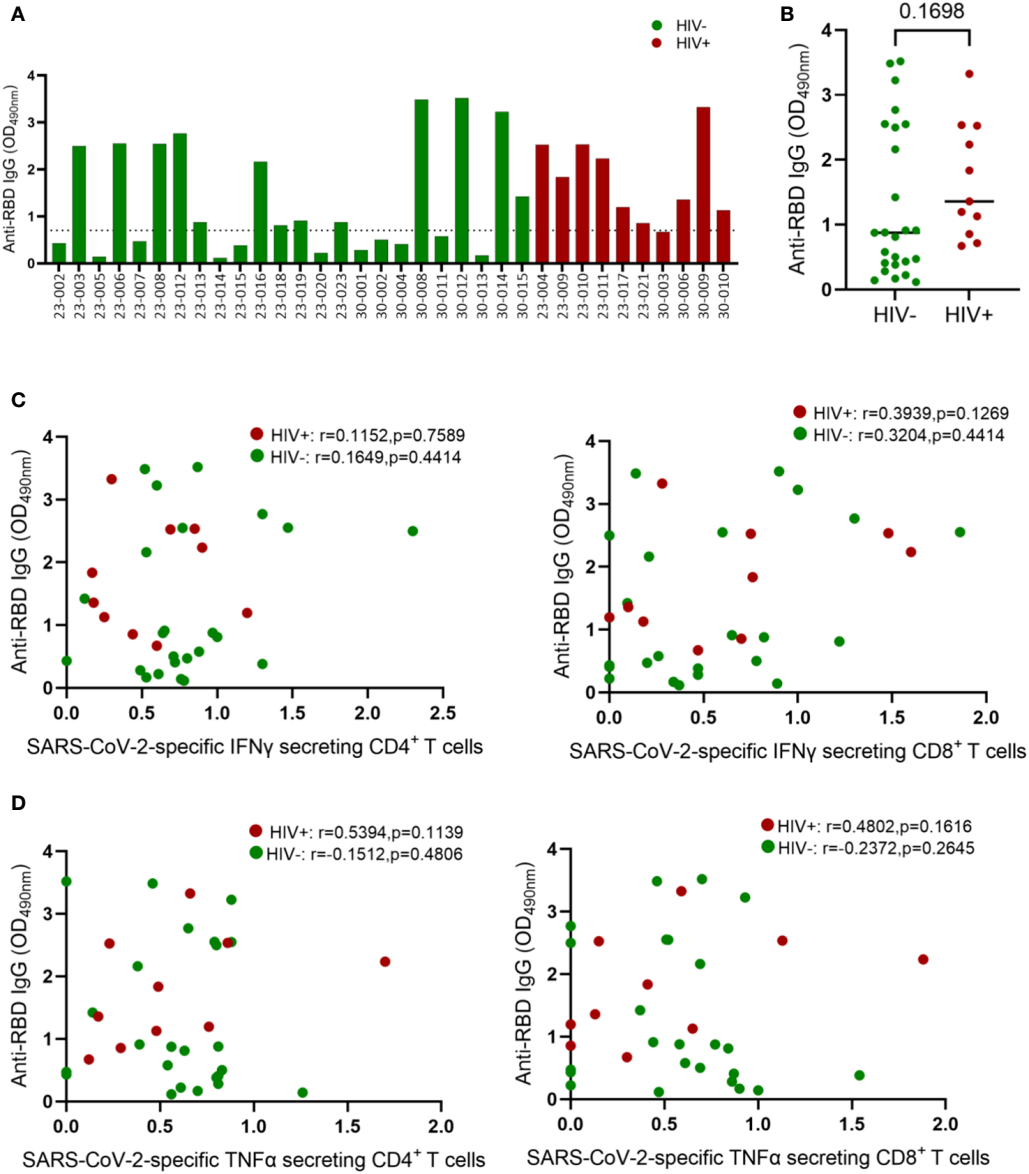
Comparison of IgG concentrations in convalescent HIV-infected and HIV-uninfected individuals. Serum samples collected between 1- and 28-days post infection were used to measure spike-specific responses by ELISA. **(A)** Comparison of anti-RBD IgG antibody OD values in convalescent HIV-infected (red bars, *n* = 10) and HIV-uninfected (green bars, *n* = 24) individuals. **(B)** Aggregate data of anti-RBD antibodies in HIV infected and HIV-uninfected individuals. **(C)** Correlation of anti-RBD antibodies and SARS-CoV-2 specific IFNγ secreting CD4^+^ and CD8^+^ T cells. **(D)** Correlation of anti-RBD antibodies and SARS-CoV-2 specific TNF-α secreting CD4^+^ and CD8^+^ T cells. The dotted line denotes OD values ≤ 0.7 that represent a negative ELISA test. ELISA tests are positive if the average OD value is > 0.7. Significance was determined by two-tailed Mann-Whitney test and the two-tailed nonparametric Spearman test was used for correlation analysis, *p*< 0.05 was considered statistically significant.

Having demonstrated the negative effects of HIV infection on T cell immunity to EHC and SARS-CoV-2, we next investigated whether HIV has similar deleterious effects on humoral immunity to coronaviruses. Firstly, we used an in-house ELISA assay to measure antibodies targeting the receptor-binding domain (RBD) of the spike (S) protein of SARS-CoV-2 because of the potential of these antibodies to neutralize the virus and therefore desirable to induce by vaccination (30–32). We screened plasma samples from 10 HIV-infected and 24 HIV-uninfected subjects (PCR-confirmed SARS-CoV-2 infection) for antibodies against the RBD antigen. A sample was considered positive if the O.D value was greater than or equal to 0.7 and negative if the O.D value was less than 0.7, as previously described (33). The ELISA results are reported as optical densities with the limit of detection set at 0.7 shown by the dotted line on the graph (**Figure 4A**). Fifty four percent (13/24) of the HIV-uninfected individuals had measurable anti-RBD IgG antibodies, whereas 90% (9/10) of the PLWH had measurable anti-RBD antibodies. The aggregate data showed no significant difference in anti-RBD antibody production based on HIV status (**Figure 4B**).

Virus specific CD4^+^ T cells responses are known to help B cell responses, but the role of effector CD4^+^ T cells responses in promoting B cell affinity maturation and antibody class switching during SAR-CoV-2 infection remain unresolved (34). Several studies have reported positive association between SAR-CoV-2 specific CD4^+^ T cell frequency and the levels of Spike specific antibodies in plasma (12). Others have shown that antibodies generated in the presence and absence of Tfh cells display similar neutralization potency against SARS-CoV-2 (34–36). Here, we investigated the connection between anti-RBD antibody levels and SARS-CoV-2 T-cell responses in both HIV-infected and HIV-uninfected donors. Our analysis revealed no correlation between RBD IgG titers and SARS-CoV-2-specific IFN-γ-secreting CD4^+^ and CD8^+^ T cells (**Figure 4C**). Similarly, TNF-α-secreting SARS-CoV-2-specific CD4^+^ and CD8^+^ T cells showed no correlation with RBD IgG titers (**Figure 4D**). The absence of correlation between antibody responses and CD4^+^ T cell responses might be attributed to our measurement of overall CD4^+^ T cell responses instead of follicular helper cells (TFH), which have been linked to anti-spike antibody responses (37, 38).

### 3.5 Antibody recognition of SARS-CoV-2 antigens and ACE2 binding inhibition by healthy control and SARS-CoV-2 convalescent plasma

**Figure 5:**
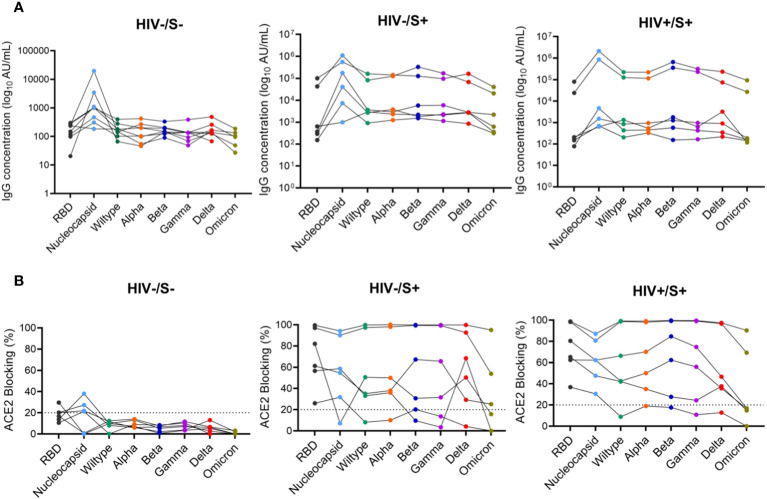
Comparison of anti-SARS-CoV-2 IgG antibodies and ACE2 blocking potential in healthy controls and COVID-19 convalescent HIV-infected and HIV-uninfected individuals. Serum samples collected between 1- and 22-days post infection were used to measure anti-SARS-CoV-2 IgG antibodies and ACE2 blocking in HIV-infected (HIV+, *n* = 6), HIV-uninfected (HIV-, *n* = 6) and healthy controls (*n* = 6) by the MSD V=Plex assays. **(A)** Summary plots of SARS-CoV-2-specific IgG antibody concentrations in the three groups. **(B)** Summary plots showing ACE2 blocking of SARS-CoV-2-specific antigens in the three groups.

Next, we used the SARS-CoV-2 MSD Multi-Spot Assay System (V-PLEX COVID-19 serology assay) to quantify binding and neutralization activity and evaluate anti-spike antibodies in plasma. Specifically, the V-PLEX COVID-19 serology assay allowed us to measure IgG antibody binding activity to multiple antigens such as RBD, nucleocapsid, wildtype, alpha, beta, gamma, delta, and omicron using multi-spot plates (**Table 3**). Twelve SARS-CoV-2 convalescent participants (6 HIV-infected and 6 HIV-uninfected) and 8 healthy controls were used for these studies based on sample availability (**Table 2**). We found that most COVID-19 convalescent participants’ IgG antibodies were able to bind all SARS-CoV-2 variants except omicron with significantly higher levels of IgG antibodies targeting the SARS-CoV-2 antigens compared to healthy donors (**Supplementary Figure 3**). The nucleocapsid and beta antigens were most targeted (**Figure 5A**). There was low-level detection of anti-nucleocapsid antibodies in one of the healthy controls, likely attributable to cross-reactive antibodies (**Figure 5A**).

**Table 3:** List of antigens and their spot assignments on the MULTI-SPOT 96-Well, 10-Spot plate (Plate 24).

Plate description	SARS-CoV-2 antigen	Antigen description
**Spot 1**	SARS-CoV-2 Spike	Wildtype
**Spot 2**	Spike (B.1.1.529; BA.1; BA.1.15)	Omicron
**Spot 3**	SARS-CoV-2 Nucleocapsid	Nucleocapsid
**Spot 4**	Spike (B.1.617.2; AY.4) Alt Seq 2	Delta
**Spot 5**	BSA	–
**Spot 6**	BSA	–
**Spot 7**	Spike (P.1)	Gamma
**Spot 8**	Spike (B.1.1.7)	Alpha
**Spot 9**	Spike (B.1.351)	Beta
**Spot 10**	SARS-CoV-2 S1 RBD	Receptor binding domain

Next, we examined the neutralization activity of plasma anti-SARS-CoV-2 Spike antibodies. We used a V-PLEX COVID-19 ACE2 neutralization assay which measures the ability of antibodies to block the binding of ACE2 to its cognate ligands. The V-PLEX COVID-19 ACE2 neutralization assay has been demonstrated to highly correlate with gold standard live microneutralization assays (14). The neutralization assay was performed on samples that had detectable antibodies by V-PLEX COVID-19 serology assay. Almost all COVID-19 participants generated significant RBD-ACE2 binding inhibition of all the variants tested and no significant difference was observed between PLWH and HIV-uninfected participants (**Figure 5B**). Generally, plasma from healthy subjects did not exhibit ACE2 binding inhibition except 3 donors who exhibited minimal binding activity (ACE2 binding inhibition of< 20%) against the nucleocapsid (**Figure 5B**). These results indicate that HIV infection has minimal impact on the quality and magnitude of antibody responses against SARS-CoV-2 infection as the SARS-CoV-2-specific antibody responses were similar between PLWH and HIV-uninfected participants. These results are similar to other studies that have shown that antibody responses to SARS-CoV-2 infection did not differ by HIV status (15).

## 4 Discussion

Enhancing our understanding of both cellular and humoral immune responses to COVID-19 within populations at higher risk of infection or severe illness is crucial for the development of next-generation COVID-19 vaccines, aiming to provide superior protection across all demographic groups. In this study, we aimed to identify the underlying immune deficiencies contributing to weakened immune responses against SARS-CoV-2 in PLWH. We focus on exploring the role of pre-existing cross-reactive responses in SARS-CoV-2 immunity, as various studies have underscored the potential benefits of cross-reactive immunity in both SARS-CoV-2 infection and vaccination (9, 17). Furthermore, the significance of cross-reactive immunity to various coronaviruses is noteworthy in the creation of panCoV T cell-inducing vaccines, designed to safeguard against multiple coronaviruses.

Our study uncovered a notable relationship between pre-existing immunity and the development of cross-reactive responses to SARS-CoV-2. To begin with, we noticed higher occurrences of HCoV-NL63 memory responses in individuals with COVID-19 compared to those who were healthy. Secondly, a strong correlation emerged between HCoV-NL63 memory responses and SARS-CoV-2 responses, suggesting that pre-existing cross-reactive immune responses are present in most individuals. Thirdly, we observed that people living with HIV (PLWH) had lower frequencies of pre-existing memory T cell responses to the endemic human coronavirus (EHC) HCoV-NL63. Fourthly, we noted that HIV infection had a more detrimental impact on cellular immune responses than on antibody immune responses to both HCoV-NL63 and SARS-CoV-2 infections. Overall, our study underscores the need to better understand of how cross-reactive responses influence vaccine-induced immune responses.

Various studies have presented evidence of pre-existing T cells recognizing SARS-CoV-2 in individuals across different geographic regions (13, 39). The idea is that cross-reactive memory T cells, stemming from previous exposure to other circulating coronaviruses, contribute to a baseline immunity against COVID-19 (40, 41). As such, the higher vulnerability of the elderly to severe COVID-19 has been linked to reduced pre-existing cross-reactive CD4+ T cell responses (17). Furthermore, a study by van Rooyen et al. (4) highlighted significant pre-existing T-cell immunity to SARS-CoV-2 in South African individuals who hadn’t previously been diagnosed with COVID-19. This immunity might be attributed to pre-existing cross-reactive immune responses to other human coronaviruses or asymptomatic infections. The study also observed that the strength of T cell responses to both SARS-CoV-2 S-proteins and N-proteins was greater in participants with a history of COVID-19 diagnosis, indicating a notable T cell response post-SARS-CoV-2 infection (4).

In our previous study, we focused on comparing individuals infected with the wildtype and beta variant, exploring cross-protection between the first wave virus and the beta variant, and examining the influence of HIV infection on cross-recognition. We demonstrated that unsuppressed HIV is linked to weakened immune responses and limited recognition of COVID-19 variants. However, the primary factors contributing to these suboptimal responses were not identified. In this current study, our focus shifted to investigating whether pre-existing immune responses to a common cold coronavirus can cross-recognize and cross-protect against SARS-CoV-2 infection. This current study suggests that the inadequate cross-reactivity of pre-existing memory responses to endemic human coronaviruses (EHCs) might contribute to the overall decline in T cell responses to SARS-CoV-2. It’s noteworthy that both studies share a common theme of assessing the impact of HIV infection on the quality and magnitude of pre-existing immunity to SARS-CoV-2 and common cold coronaviruses. These findings are consistent with earlier studies that indicate HIV infection, even when managed with effective antiretroviral therapy, is characterized by chronic immune activation, tissue inflammation, and exhaustion (42, 43).

It has been reported that the severity of clinical disease in COVID-19 patients is associated not only with significant changes in the innate immune system but also with a marked alteration of both humoral and cellular immunity, encompassing SARS-CoV-2–specific and overall T cell function (44). Our immunological analysis revealed that both SARS-CoV-2 and EHC responses were more focused on the CD4 arm of the cellular immune system rather than the CD8 arm, which is consistent with our prior study and other reports (12, 17, 20, 45). Furthermore, HIV infection had a more profound effect on CD4^+^ T cell responses compared to CD8^+^ T cell responses and antibody responses. It is not clear why SARS-CoV-2 induces stronger CD4^+^ T cell responses. One potential explanation is that the most immunogenic SARS-CoV-2 antigens are more readily recognized by CD4^+^ T cells than CD8^+^ T cells. Nevertheless, further studies are required to comprehend this phenomenon.

Prior research has demonstrated similar antibody titers against S1 and N proteins of SARS-CoV-2 in individuals with mild COVID-19, regardless of their HIV infection status (46). Other studies have also indicated that there are no discernible differences in antibody responses during the 6-month period following mild COVID-19 based on HIV status. The magnitude, progression, and persistence of anti-SARS-CoV-2 IgM, IgG, and IgA antibodies, as well as neutralization strength, appear to be consistent in PLWH who have well-managed HIV infection (15, 46, 47). In line with these findings, this study reveals that PLWH have comparable levels of neutralizing antibodies in the form of anti-SARS-CoV-2 IgG, compared to individuals without HIV. Additionally, a subset of participants, both HIV-infected and HIV-uninfected, displayed robust neutralization abilities despite having low titers of anti-SARS-CoV-2 binding antibodies. This might be attributed to neutralizing antibodies targeting different viral epitopes and/or elevated levels of non-neutralizing antibodies, or the influence of other antibody isotypes in the neutralizing responses (46).

This study indicates that PLWH have diminished T cell responses to both an endemic human coronavirus (EHC) and SARS-CoV-2. This suggests a potential susceptibility to SARS-CoV-2 infection, and it’s plausible that their responses to infections and vaccines might be less robust. Nonetheless, it’s important to note that the study has certain limitations, notably a small sample size and the utilization of a cross-sectional design, which restricts the broader generalizability of these observations. To validate these intriguing findings, a more extensive longitudinal analysis of specific T cells and antibodies will be necessary.

In conclusion, our study reveals that the decrease in SARS-CoV-2 specific responses T cell in PLWH may be attributable to reduced frequencies of pre-existing cross-reactive responses, heightened T cell activation and systemic inflammation.

## Data availability statement

The raw data supporting the conclusions of this article will be made available by the authors, without undue reservation.

## Ethics statement

The studies involving humans were approved by University of KwaZulu-Natal Institutional Review Board (approval BREC/00001275/2020) and the National Health Research Authority and University of Zambia Biomedical Research Ethics Committee (REF. No. 1648-2021). The studies were conducted in accordance with the local legislation and institutional requirements. The participants provided their written informed consent to participate in this study.

## Author contributions

TNg’: Data curation, Formal analysis, Investigation, Writing – original draft, Writing – review & editing, Methodology. VM: Investigation, Writing – review & editing. TNk: Formal analysis, Investigation, Writing – review & editing. JM: Formal analysis, Investigation, Writing – review & editing. MM: Formal analysis, Investigation, Writing – review & editing. CM: Investigation, Writing – review & editing. WK: Writing – review & editing, Conceptualization, Funding acquisition, Project administration, Resources. FK: Project administration, Writing – review & editing, Data curation. MM: Project administration, Writing – review & editing, Resources. KK: Writing – review & editing, Data curation, Project administration. KJ: Writing – review & editing, Formal analysis, Investigation, Resources. WH: Resources, Writing – review & editing, Project administration. AS: Data curation, Funding acquisition, Project administration, Resources, Writing – review & editing. ZN: Conceptualization, Data curation, Formal analysis, Funding acquisition, Investigation, Project administration, Resources, Supervision, Validation, Writing – original draft, Writing – review & editing.
