# Supplementary material for: Low pre-existing endemic human coronavirus (HCoV-NL63)-specific T cell frequencies are associated with impaired SARS-CoV-2-specific T cell responses in people living with HIV
Source: Front Immunol. 2024 Jan 26;14:1291048. doi: 10.3389/fimmu.2023.1291048 (PMC10853422; doi:10.3389/fimmu.2023.1291048)
Supplement: Supplementary file 1 [file DataSheet_1.pdf]

Supplementary Material

1 Supplementary Figures

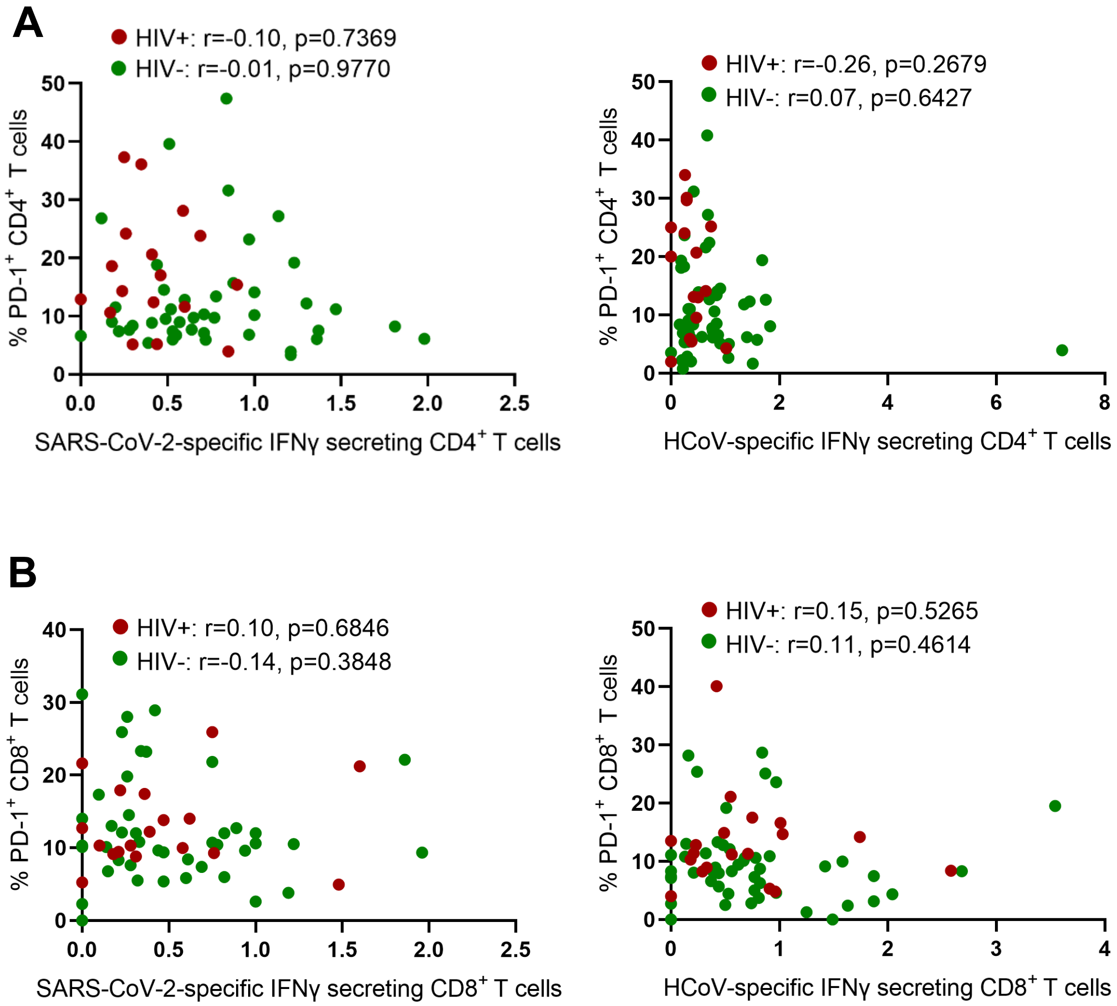

Supplementary Figure 1. Comparison of the exhaustion profile of SARS-CoV-2- and HCoV-specific specific $CD4^+$ and $CD8^+$ T cells in COVID-19 convalescent HIV-infected and HIV-uninfected Individuals. Blood samples collected between 1- and 28-days post infection during the second (beta) and third (delta) waves were used to detect activated and exhausted T cells in HIV-uninfected (HIV-, $n = 46$) and PLWH (HIV+, $n = 20$. (A) Correlation of T cell exhaustion and virus-specific $CD4^+$ T cells based on the expression of IFNγ in HIV-infected and HIV-uninfected individuals. (B) Correlation of

T cell exhaustion and virus-specific CD8+ T cells in HIV-infected and HIV-uninfected individuals. Spearman test was used for correlation analysis, $p < 0.05$ was considered statistically significant.

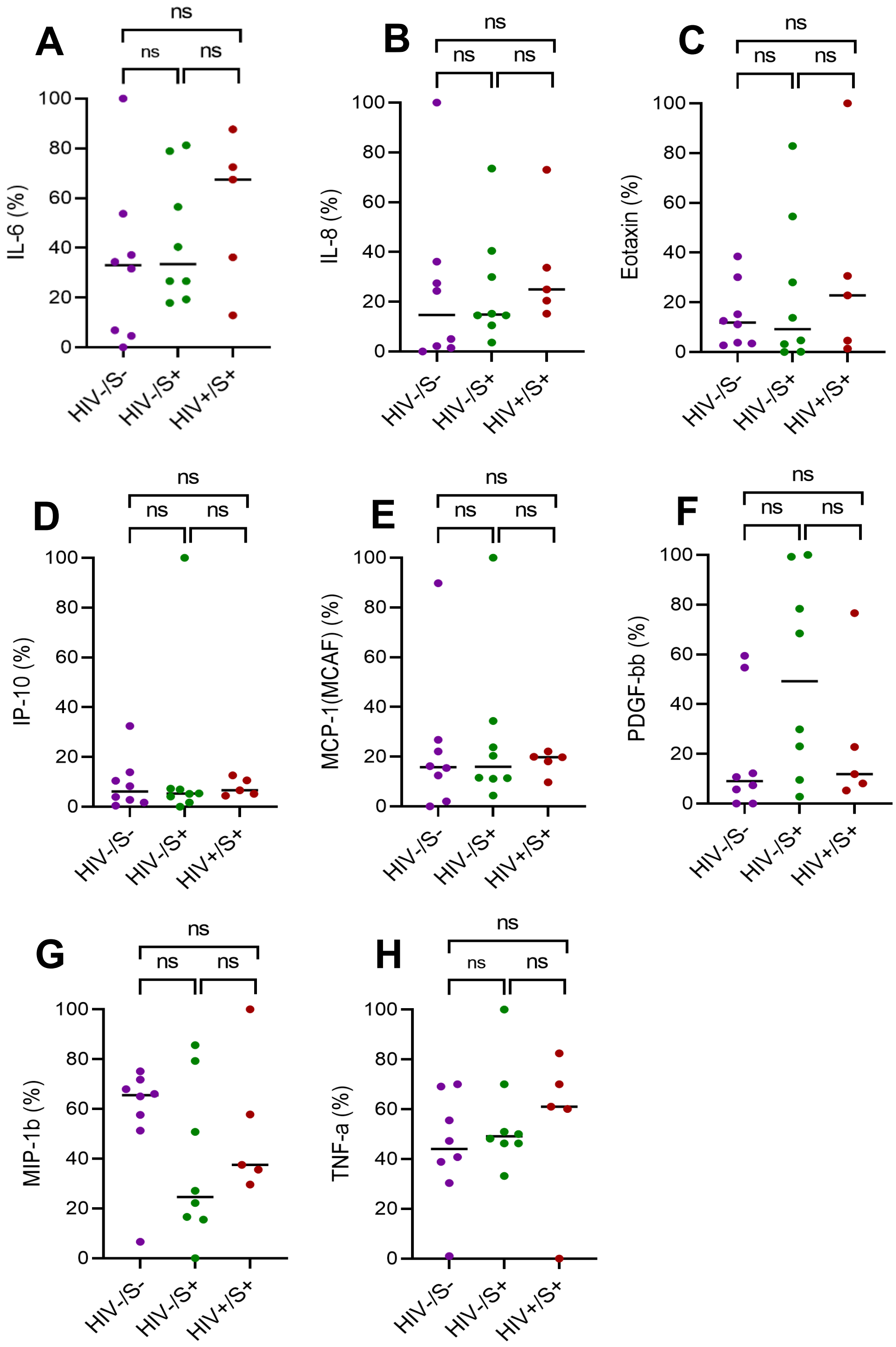

Supplementary Figure 2. Comparison of plasma cytokine and chemokine levels in cells in convalescent HIV-infected and HIV-uninfected individuals and healthy controls. Serum samples collected between 1- and 28-days post infection were used to measure cytokine and chemokine levels

by the Bio-Plex assay. (A) Figures show normalized cytokine and chemokine levels (in percentages) in convalescent HIV-infected (HIV+/S+, *n* = 5) and HIV-uninfected (HIV-/S+, *n* = 8) individuals and healthy controls (HIV-/S-, *n* = 8). Summary plots of (A) IL-6, (B) IL-8, (C) Eotaxin, (D) IP-10, (E) MCP-1(MCAF), (F) PDGF-bb, (G) MIP-1b, and (H) TNF-a. Significance was determined by two-tailed Mann-Whitney test, $p < 0.05$ was considered statistically significant.

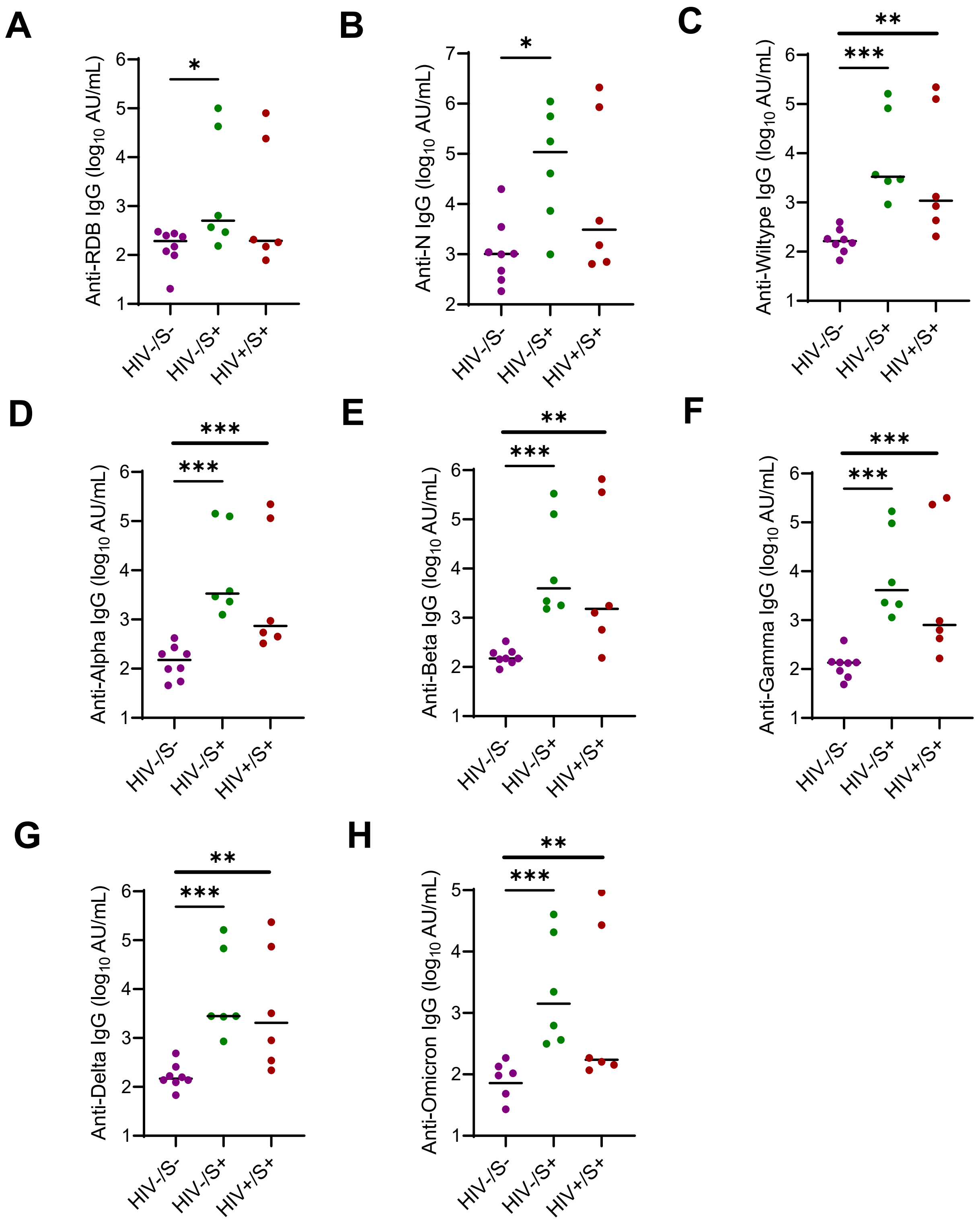

Supplementary Figure 3. Comparison of SARS-CoV-2-specific antibody responses in healthy controls and COVID-19 convalescent HIV-infected and HIV-uninfected Individuals. Serum samples collected between 1- and 22-days post infection were used to measure anti-SARS-CoV-2 IgG antibodies and ACE2 blocking in HIV-infected (HIV+, *n* = 6), HIV-uninfected (HIV-, *n* = 6) and healthy controls (*n* = 8) by the MSD V=Plex assays. Summary plots showing IgG antibodies against (A) RDB, (B) Nucleocapsid (N), (C) Wildtype, (D) Alpha, (E) Beta, (F) Gamma, (G) Delta, and (H) Omicron. Significance was determined by two-tailed Mann-Whitney test, $p < 0.05$ was considered statistically significant. Data are expressed as median log10 arbitrary units (AU) per mL.
